# Ethical considerations for the use of consumer wearables in health research

**DOI:** 10.1177/20552076231153740

**Published:** 2023-02-01

**Authors:** Anna Sui, Wuyou Sui, Sam Liu, Ryan Rhodes

**Affiliations:** 1 School of Health Studies, Western University, Canada; 2 School of Exercise Science, Physical and Health Education, 8205 University of Victoria, Victoria, Canada

**Keywords:** Digital health, general, eHealth, general, electronic, general, machine learning, general, technology, general, behaviour change, lifestyle, exercise, lifestyle, lifestyle, lifestyle, wearables, personalized medicine

## Abstract

**Background:**

The UN's High Commissioner's request for a moratorium on the use and adoption of specific Artificial Intelligence (AI) systems that pose serious risk to human rights, this commentary explores the current environment and future implications of using third-party wearable technologies in research for participants’ data privacy and data security. While wearables have been identified as tools for improving users’ physical and mental health and wellbeing by providing users with more personalized data and tailored interventions, the use of this technology does not come without concern.

**Objective:**

Primarily, as researchers, we are concerned with enmeshment of corporate and research interests and what this can mean for participant data.

**Methods:**

By drawing on specific sections of the UN Report ‘The right to privacy in the digital age’, we discuss the conflicts between corporate and research agendas and point out the current and future implications of the involvement of third-party companies for participant data privacy, data security and data usage. Finally, we offer suggestions for researchers and third-party wearable developers for conducting ethical and transparent research with wearable tech.

**Conclusion:**

We propose that this commentary be used as a foothold for further discussions about the ethical implications of using third-party wearable tech in research.

## Introduction

We cannot afford to continue playing catch-up regarding AI – allowing its use with limited or no boundaries or oversight and dealing with the almost inevitable human rights consequences after the fact.^1^

On September 15, 2021, the United Nations High Commissioner for Human Rights, Michelle Bachelet, called for a moratorium on the use and adoption of Artificial Intelligence (AI) systems that pose a ‘serious risk to human rights’.^2^ A similar moratorium was called by the UN for the use of lethal autonomous robots as they represent a form of technology where ‘little may be known about the potential risks of the technology before it is developed, which makes formulating an appropriate response difficult; but afterwards, the availability of its systems and power of vested interests may preclude efforts at appropriate control’.^3^ For machine learning technologies in particular, the surveillance, profiling and monitoring of people in public spaces have been criticized for their potential to compromise human rights and result in (often unforeseen) negative consequences on welfare.^2^ The high commissioner suggested that we need proper safeguards in place to protect the lives and livelihoods of the people involved before further tech development can happen. While the moratorium concerned surveillance and ‘biometric’ AI, including real-time facial-scanning technologies, other large aggregates of data for commercial purposes pose similar risks to user privacy and data safety.

One such aggregate of data lies within wearable technologies or wearables. Wearables represent a promising tool for health research^4^ and interventions.^5^ However, consumer wearable tech (e.g. FitBits, Apple Watches, Xiaomi) exposes their users to heightened surveillance, and may involve sacrifices to data privacy and compromises to data security. In a recent security breach, over 61 million fitness tracker records from Apple and Fitbit were exposed online, compromising the data privacy of their users.^6^ As digital technologies, like consumer wearables, continue to shape our society and behaviours, critical and concurrent evaluation of the ethical impacts these devices may have on their users (e.g. research participants) is imperative. Accordingly, the call for a moratorium provides an important opportunity to review the current use of data aggregation, storage and usage practices when using consumer wearables in research. Closer examination into the relationships between the participant, researcher and third-party developers and how these may compromise participants’ safety, privacy and autonomy are warranted.

Within research contexts, wearable technologies are nothing new. Since the beginning of the wearable research boom in the early 2000s, publications covering wearables have increased nearly ten-fold in the past decade alone, according to Scopus (i.e. 1488 papers and 11,358 papers, respectively). Pedometers and accelerometers have been used in health and kinesiology studies both to monitor and improve physical activity in participants.^7,8^ Heart rate monitors and continuous glucose monitors provide near-constant feedback on physiological markers. While these non-smart wearables (i.e. wearables without the ability to use the Internet of Things) still rely on algorithms for collection and analysis of participant data, these are often openly available or validated against another standard measure and supported by peer-reviewed work.^9^ Further, collected participant data remains solely with the researcher when using non-smart wearables. However, this is not the case with newer, more consumer-oriented tech. The use of consumer wearable tech for research is attractive for several reasons. These devices tend to be cheaper to purchase (e.g. $250 ActiGraph GT3X accelerometer^10^ vs. $99 Fitbit Inspire 2^11^), are designed with heavy focus on a positive user experience and come with a variety of features and customizations offering easy access to technical support for users (e.g. researchers, participants). However, these devices also pose unique challenges for researchers. Participant information often gets stored and analysed by third parties using ‘black box’ algorithms.^12^ Black box algorithms can be understood in terms of input and output, however, the inner working of the algorithm are unknown to researchers (and often the company themselves,^13^ and can also change on a whim – both of which can compromise the reliability of a research trial.

Specifically, several concerns arise from the use of consumer wearables. First, the kinds of data collected and what the data will be used for are often outside the control of the researcher.^14^ Second, the use of third-party tech embeds company commercial interests within research goals, which can impact the research process. Finally, participant data security can be compromised through data transfers and storage. In the following section, we discuss these concerns and propose several considerations for researchers working on consumer wearable tech.

### Third-party involvement in research practices

**Figure 1.**
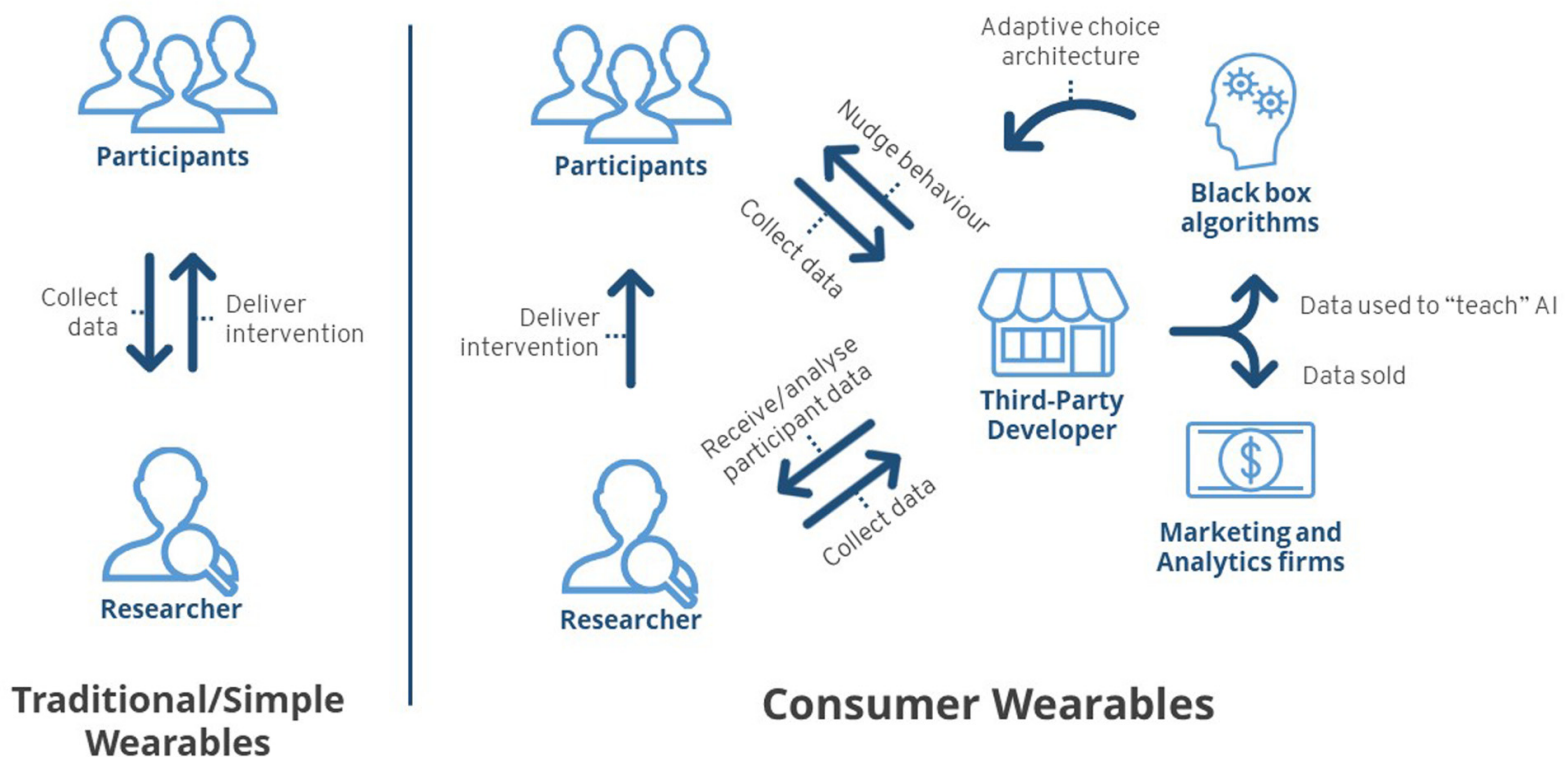
Data transaction differences between traditional/simple wearables and consumer wearables.

The use of third-party wearable technology introduces a third actor into the researcher/participant relationship (see Figure 1). With non-smart wearables, such as pedometers and accelerometers, the role of the company was limited to the sale of the device and/or analysis software. Data collected from the device or analysed by the software was confined to the researcher-participant relationship; the third-party provider was not sent this data but acted as a mediator between the participant and the researcher.

By comparison, the use of consumer wearables embeds the third-party company into the research process through the continuous transfer of data from participants (via the wearable device) to the company. Data collected from participants’ wearables no longer involves solely the researcher, as collected data are sent to the company as part of the data collection/analysis process. These data practices may even extend to other, less-defined, third-party companies.^15^ As such, consumer wearables facilitate an ongoing relationship and data exchange with the researcher, participant and company. These data, often collected without the knowledge of the wearable user, ‘largely shielded from public scrutiny’^2^^(p4)^, can then be used for a multitude of different purposes. Notable examples include selling this data to third-party vendors or marketing companies or using aggregated data as learning datasets for improving black box algorithms to influence consumer behaviour (discussed later) more effectively.

In contrast to the researcher, whose use of participants’ data is clearly outlined and analysed, data usage by companies may be less transparent or accessible.^16^ When options for data usage are provided to the user, they are difficult to locate and decipher.^17^ While effort has been made by some companies to make data collection more transparent and accessible,^18^ these policies ultimately render both the researcher and the participant at the mercy of the company's data policies. This is problematic, as discussed later, largely due to the conflicting interests of the company and researcher, but also due to imperfect practices in data collection, data storage and transfers.

### Data collection, usage and privacy by third parties

Furthermore, since the computational knowledge and power over AI systems tends to be held by private companies, these arrangements often mean that private companies gain access to data sets containing information about large parts of the population. This raises privacy concerns as well as concerns about how historic bias embedded in data will affect the decision-making of public authorities.^2^^(p8)^

Researchers, by virtue of the transparency and replicability of the research process, are upheld to explicit and strict guidelines of what data can be collected during a study and how the data will be analysed. These practices are communicated to the participants through a Letter of Information (LOI) which is approved by an institutional ethics board (or equivalent) before a study can begin. These practices are in place to safeguard participants’ privacy, as excessive data collection may place participants at a greater risk for privacy violation. Most consumers place high importance in controlling what data is collected about them,^19^ though this perception varies among individuals.^20^ Regardless, the onus is upon the researcher to minimize risk (e.g. breaches in data privacy) as much as possible. As such, researchers must also justify the collection of certain types of data (e.g. medical information, potentially identifying information).

In contrast to the parsimonious data collection methods of researchers, companies tend to be motivated to collect as much information as possible. Data collection practices (often outlined in Privacy Statements) are conveyed in a convoluted ways whereby the language used is inaccessible to the average person. This may lead to data collection practices that, if otherwise clearly communicated, many users would likely decline.^21^ Indeed, this practice is overwhelmingly effective, with one study estimating that 97% of people indicate agreement to a privacy policy that should have taken approximately 30 min to read, in just 51 s.^21^ Opting out of these data collection policies is problematic, as the mere operation of some consumer wearables is predicated on the free, ‘consensual’ transference of all data from the device to the company.^15^ While some major corporations such as Apple are moving towards better data security for their users, such as the new Mail App masking the user's IP while they use it,^22^ increased data privacy practices are not industry standard. Notably, user data breaches are not deliberate, but rather occur as a consequence of malware attacks, human error and hacking.^23^

Extensive data aggregation by companies serves several purposes. First, the data can be used to analyse the demographics of the wearable user base to identify who the consumers of the product are and what kind of behaviour(s) they perform, which by extension, can be sold to analytics and marketing firms. In this way, wearable companies act as ‘data brokers’, acquiring, merging, analysing and sharing personal data with ‘countless recipients’.^2^^(p4)^ Second, data can be used to teach AI. More specifically, datasets of ‘unprecedented proportions’^2^^(p4)^ are used to train algorithms how to better predict, influence and adapt to consumers. The complexity, and often propriety, of these algorithms renders cross-examination of the inner workings and decisions made by these systems inaccessible, or opaque, to the participant, researchers, and often the developer themselves. Due to the opacity of the processes, functions and models that inform this ‘black box’ algorithm decision-making, outputs cannot be dissected or explained. Further, the performance of an algorithm is highly contingent on the dataset(s) it is trained on.^24^ Taken in tandem, black box algorithms can result, and have resulted, in replicating many of the human prejudices and biases that are present in training data.^24^ When considering consumer wearables specifically, ‘black box’ algorithms risk making discriminatory conclusions based on sensitive health data^25^ or inaccurate data due to hardware limitations,^26^ and can encroach upon individuals’ previously private spheres, such as employment and health insurance.^2^^(p8)^ As the optimal or ideal outputs of these algorithms are designed to align with the developer's (i.e. company's) own interests, cognisance for what data is taken, who has or is given access to this data, and what it will be used for, is paramount for researchers using consumer wearables.

### Commercial vs. research goals

Many inferences and predictions deeply affect the enjoyment of the right to privacy, including people's autonomy and their right to establish details of their identity. They also raise many questions concerning other rights, such as the rights to freedom of thought and of opinion, the right to freedom of expression, and the right to a fair trial and related rights.^2^^(p5)^

Often, the goals of researchers and those of companies that develop consumer wearables differ. While researchers may prioritize the pursuit of science and knowledge, for-profit companies may need to balance scientific endeavours and their commercial interest^27^ (e.g. profitability, sustainability). Within the context of wearable tech, these commercial influences are often pursued through the interest of improving health. This is often accomplished through variable ‘choice architecture’^28^ or the ways in which choices can be presented to users through the app/software they are using and shape user decisions by swaying their choice one way or another. A review of the behaviour change techniques (BCT; i.e. established method used to change behaviour^29^) of consumer wearable technologies highlights the extensive number of BCTs used by these devices^30^ – well above the number used by comparable in-person interventions or other digital interventions.^31^ While these influences can, and often are, beneficial to the user's health, the primary goals of these architecture, and associated ‘invisibility’ and adaptability warrant deeper inspection.

This ‘invisible influence’^32^ is especially concerning when commercial interests fixate on disclosure and collection of more data or the purchasing of a product. Conflicting interests for choice architecture arise from this seemingly seamless experience in using tech. In this sense, we are no longer aware of the ways we engage with the world through technology since the seams of using it have been largely removed.^32^ Hence, the choice architecture shapes our decisions, often without our recognition of the process, which, Susser suggests, compromises user autonomy since their decisions are shaped without their knowledge.^32^ For example, Garmin watches – which function as pedometers, accelerometers and have other tracking functions that make them popular for runners – suggest articles to the wearer if they fail to meet certain metrics (e.g. activity goals) to ostensibly help improve their health and well-being, while tacitly promoting other Garmin services or products within these articles.^33^ This type of ‘adaptive choice architecture’, which uses the participants’ own data to influence their behaviour, is especially problematic in research settings. Invisible influence from the use of consumer wearables heralds the potential for corporate interest to directly shape research outcomes by influencing participant interactions with their wearables. Similar concerns about the influence of AI over consumer decisions (e.g. prediction products) have been previously voiced.^34,35^ Problematically, while choices to opt out of these services exist, they are not the default option, which may dissuade many from doing so (see ‘Status Quo Bias’^28^). Aside from the implied violations to participants’ right to freedom of thought and opinion, the black box nature of these technologies obfuscates key elements of the research process (e.g. mechanism of intervention), particularly for behaviour science.

### Data security concerns with third-party uses

Data breaches have repeatedly exposed sensitive information of millions of people. Large data sets enable countless forms of analysis and sharing of data with third parties, often amounting to further privacy intrusions and incurring other adverse human rights impacts.^2^^(p4)^

Similar to participant safety and privacy, clear and rigorous guidelines for how participant data is stored (e.g. where/how long data is stored, deidentification of data, who has access to the data, data destruction protocols) are both adhered to by the researcher and explicated to the participant. By comparison, companies could, but often do not need to, follow the same rigorous guidelines (e.g. HIPAA^36^). Rather, the data storage guidelines for companies tend to be a lot murkier. For example, data becomes more accessible once it falls under the jurisdiction of the country where physical servers are located. Google's (like many other tech giants) servers are stationed in Ireland, which has been criticized for creating a potential ‘regulatory safe zone’ for companies within Europe.^37^ As such, data becomes much easier to access, even in legal ways, once it is stored in overseas servers.^38^ Importantly, many of the contracts that the user agrees to while using wearable tech (e.g. end-user agreement, terms of service) fall outside of IRB jurisdiction since these agreements happen between the user (i.e. participant) and the third-party company.^39^

Additionally, commercial data storage often involves the transfer of data between several servers (e.g. marketing companies, analytics firms, etc.) located in various geographical locations. Different safety standards for data storage may then apply, inadvertently compromising data security. This means that data security is limited by the weakest link. Security breaches across companies are neither uncommon nor a new phenomenon^40^ and have led to several high-profile scandals involving the loss of user data.^41^ Additionally, analytics firms may use the data in unintended ways such as when Strava released a heat-map detailing American military placement around the globe based on the geolocation data they’ve acquired from the personnel's Fitbits and other fitness trackers.^42^

For researchers using consumer wearables, conflicting data security practices between themselves and the wearable company present as a threat to their participants’ privacy, identity and perhaps even autonomy. Since data collected from consumer wearables is sent to the company as a function of using the device, researchers can do little to guarantee the safety of participant data.

Finally, the ways in which companies dispose of user data are unclear.^43^ This raises questions about when (or even whether) participant data is destroyed following cancellation of an account or subscription – and if so, how. The ambiguity around data security and data disposal inherently prevents users from making an informed choice about whether or not they consent to the data collection practices, which as previously mentioned, are often required for the use of the wearable itself.

### Suggestions for researchers

In this final section,^2^ we provide several suggestions for researchers using wearables in their projects. We position these suggestions based on the ‘State-business nexus’ mentioned in the UN report, whereby the State ‘is an important economic actor that can shape how AI is developed and used’, and the State-business nexus is ‘a close nexus between a State and a technology company require[s] dedicated attention’.^2^^(p13)^ We supplant the role of the State with the role of the researcher and propose that the following suggestions are only feasible through a researcher-business nexus, whereby researchers oversee the development and deployment of AI systems through demanding and assessing information about the accuracy and risks of an AI application.

First, we echo the suggestions made by the UN on the use of AI: radical transparency is needed from third-party providers about how the data is being collected, analysed and used. While this is often mentioned in Privacy Agreements, we argue that a more accessible version is needed. This transparency should also extend to the outcomes taken by consumer wearables. Any outcome that cannot be fully explicated by a wearable device/algorithm should be rejected. In line with this, standardized and transparent reporting guidelines for the use of these devices (akin to CONSORT^44^ or PRISMA^45^ guidelines) should be developed, ideally in partnership with consumer wearable developers. For example, companies may provide an itemized list to researchers of the variables a consumer wearable is capturing (e.g. heart rate, location, accelerometry), as well as what this data can potentially be used for (e.g. marketing, third-party analytics, algorithm training).

For researchers, the benefits and risks of using wearables in their research need to be weighed. If consumer wearables are to be used, the risks for participant privacy need to be disclosed to future participants in the LOI, in addition to providing study details. Some researchers have addressed these concerns by providing their participants with two LOIs: one for the study and one for how the participant data will be handled by the third parties. Alternatively, anonymous accounts could be made for participants when using these wearables. However, given that the validity of these algorithms typically relies upon the input of accurate user information,^32^ the intended utility of these devices may be compromised without some degree of identifying information.

Another valuable addition to wearable tech would be ‘seamful’ design.^46^ As technology becomes more advanced, our interactions through it become seamless and users become unaware that they are even using tech (and that tech is shaping their decisions). Adding ‘seams’ to the tech-user experience will remind users that their experiences and decisions are mediated by technology. For example, some websites will prompt the viewer that cookies are being collected and will have the opportunity to opt out of having their cookies monitored. While few viewers will opt out, this added seam reminds the viewer that their data is currently being collected.

The major limitation to these suggestions is that, unlike the State, researchers are rarely an economic actor in the third-party/researcher relationship. Hence, researchers have little to provide as incentive for third parties to change their practices or work together with researchers to develop ethically sound ways to collect/store/analyse data. Further, researchers lack the legislative power that is reserved for the State and hence, can set no consequences or statutes for the ways in which third-party developers conduct their data aggregation practices, even within the narrow context of research. This lack of financial and legislative power places researchers in a precarious position, whereby they do not have capacity to develop their own comparable wearable device, and hence must rely on consumer wearables (and associated data practices). Ultimately, this places researchers at the whim of both the State itself and third-party developers to conduct their research.

## Conclusion

While wearables provide an exciting avenue for health and healthcare research, several ethical considerations need to be made regarding their use, especially when using third-party consumer tech. First and foremost, third-party developers are under vague and little oversight in terms of data aggregation, storage, analysis and transfer practices. These practices are guided by competing primary interests of the two actors, wherein companies are generally concerned with prioritizing profit. As such, where researchers adhere to clearly defined ethical guidelines for how participant data is handled, companies tend to aggregate as much data as possible for the purposes of marketing research, sales and AI training. This delineation between research and commercial interests raises concerns for participant data privacy and security, especially given the risk of data breaches and data leaks increases as a function of how many hands the data is passed to. For researchers considering the use of consumer wearables, explicating these risks to potential participants through accessible privacy agreement language and through letters of information is paramount. Further, collaboration with tech companies to create additional seams within the user experience and promote transparency in what data is collected and what it may be used for should be pursued. However, we recognize the limited agency that researchers have may have in this discussion. As such, this commentary is meant to serve as a foothold in the discussion of best research practices and how these may evolve as more third-party wearable tech embeds itself into research practice and process.
